# AMREF's evidence in advancing the health of women and children

**Published:** 2012-12-25

**Authors:** Teguest Guerma

**Affiliations:** 1 Director General, African Medical and Research Foundation (AMREF)

**Keywords:** AMREF, mother and child health, HIV, tuberculosis, malaria, Africa

## Editorial

Health development in Africa, and specifically improved health for mothers and children, needs to be based on tested approaches with demonstrable results. In light of the increasing health investments currently being made in Africa, generating and documenting evidence will not only increase value for invested resources but also provide a basis for programme scale-up and influence policy and practice change. The African Medical and Research Foundation (AMREF) works with African communities to ensure that these investments provide sustainable results.

AMREF's vision is lasting health change in Africa. AMREF recognises that providing the right knowledge to the right people (policy makers, health development practitioners and communities) and in the right format is critical to strengthening health systems and improving health outcomes. In April 2012, the First Biennial AMREF Programme Meeting (BAPM) was held with the aim of sharing and maximising the impact of explicit and tacit knowledge, including experiential knowledge generated from AMREF's programmes. The organisation further sought partnership with the Pan-African Medical Journal to ensure that selected papers from this meeting were documented as a journal supplement. It is hoped that this documentation and knowledge sharing will contribute to acceleration and expansion of health sector gains attained from AMREF's programmes.

This supplement contains papers written by AMREF staff and partners working in various programme Strategic Directions: Making pregnancy safe and expanding reproductive health; Reducing morbidity and mortality among children; Scaling up HIV, TB and Malaria responses; and Preventing and controlling diseases related to water, sanitation and hygiene (WASH). The papers document the organisation's experiences in working with some of Africa's poorest, remotest and most vulnerable communities. Unique health problems such as trachoma among pastoralist communities, the jigger menace in highland regions and behaviour change among sex workers are among issues addressed by these papers.

Based on experiences from five countries, AMREF shares our achievements and lessons learnt, both in scientific and best practice paper formats. I am optimistic that the evidence shared through the articles published in this supplement will contribute to the search for solutions to Africa's health challenges. We thank all those whose efforts have yielded this useful output.

